# Wellbeing and mental health during the COVID-19 outbreak

**Published:** 2020-09-01

**Authors:** Julian Eaton

**Affiliations:** 1 Mental Health Director: CBM Global & Assistant Professor: London School of Hygiene & Tropical Medicine, UK.

**Acknowledging distress is often the first step to recovery. By recognising this – and putting simple measures in place to support the population, patients and staff members – health services can contribute to greater resilience and capacity to recover following major epidemics and other emergencies.**

**Figure:**
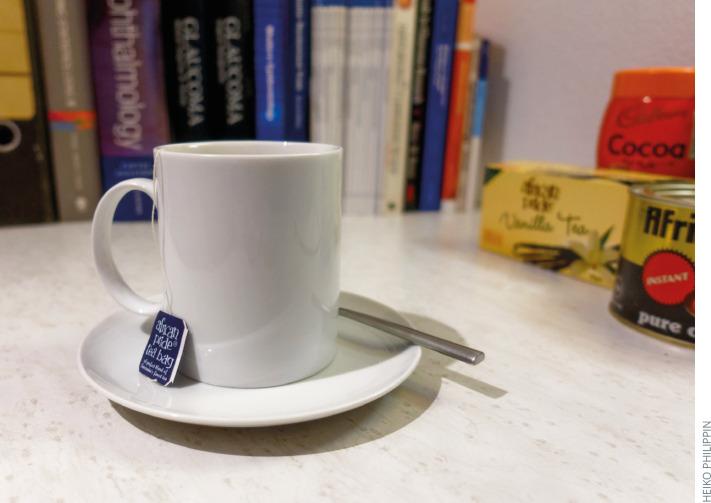
Taking time out for something as simple as a cup of tea with colleagues can help health workers to cope better with stress.

Since the beginning of 2020, it has become clear that the COVID-19 pandemic is going to have a long-term impact; not only on those directly affected, but also on the global economy and the systems that meet the health, education, social protection and other basic needs of populations. It is also likely that the pandemic will set us back in terms of achieving the Sustainable Development Goals. However, for most of us, the impact on our own lives, and on the lives of those around us, is the immediate and most pressing concern. For people who are already near the poverty line, and living without financial or other reserves, the impact is more severe. People who are marginalised due to health problems or disabilities, especially in lower-income countries, often face additional barriers to getting the care and support they need.

## Addressing mental health in communities and services

At times of disruption, change and uncertainty, it is natural for us to worry, and this can cause problems not only in feelings of anxiety and concern, but sometimes can also affect how well we function in our daily lives, our workplaces and our relationships. Increased irritability, emotional exhaustion, exacerbation of pre-existing conditions, poor concentration and poor sleep may be examples of such mental challenges.

During the COVID-19 pandemic, it is good to ensure that mental and physical wellbeing are a component of our response in the health services, so that we can minimise distress and prevent people developing more significant mental health problems, such as depression or anxiety.^1^ In addition, it is important that we care for our own mental health so that we can cope with what may be a very stressful situation and can continue to support the people around us.

Fortunately, there are good evidence-based recommendations on what to do to reduce the negative impact of the crisis and promote wellbeing. The dedicated Mental Health Innovation Network COVID-19 page has a list of resources on how to support different population groups during the COVID-19 virus outbreak: **https://bit.ly/MHINcov**

## Recommendation 1: Support people in the community

Most people cope well during stressful times by turning to those around them – their friends, families and communities – for practical and emotional support that supports their resilience: their ability to withstand stress and bounce back from difficult experiences or events. This healthy and effective means of getting help is more challenging during an epidemic where populations are advised, or even ordered, to keep physically distant or to practice isolation and quarantine.^2^

### What you can do

- Promote communication between people to minimise social isolation. This might be via telephone or online contact, or through interaction that follows safe practice in order to avoid the transmission of infection. There are also some good basic strategies that can help people to cope with the stresses of self-isolation and with their concerns or worries about COVID-19 in particular (see panel on page 36).
- Ensure that people who might be alone, isolated or marginalised are not neglected; for example, check that people who are disabled (e.g. with visual impairment) are able to receive communication on how to avoid the disease and that their basic needs are met, e.g., if they are unable to go out for shopping.
- One of the main reasons why people with disabilities (such as blindness) or diseases (such as trachoma) develop mental health problems is the stigma, discrimination and social exclusion that they experience from the people around them. An effective way to reduce this experience and promote wellbeing is to directly address stigma with community awareness programmes and to facilitate peer support. The neglected tropical diseases (NTD) website **infoNTD.org** has many helpful resources, including screening tools and stigma guidelines: **https://bit.ly/NTDwell**

**How to look after your own health and wellbeing^1^,^5^**

- Feeling under pressure is a likely experience for you and many of your colleagues. It is quite normal to be feeling this way in the current situation. Stress and feelings associated with it are not a sign of weakness, but a normal reaction to difficult times, and can be managed.
- Follow health advice, especially about avoiding the risk of getting infected or passing on the virus (e.g., wash your hands and distance yourself from others in public).
- Stay informed, but don’t immerse yourself in too much negative news. For example, only follow trusted and respected news sources. Seek information at specific times, once or twice a day.
- Maintain a healthy lifestyle: eat well, sleep well and exercise. Don’t resort to negative coping mechanisms such as smoking or drinking too much.
- Take time out if you need to. If a situation is very stressful, try to remove yourself from it.
- Find trusted people to talk to, such as friends or colleagues. It can be helpful to speak to a counsellor, if available, if those around you cannot help.
- The World Health Organization has produced an excellent, well-tested self-help guide for dealing with stress – visit **https://bit.ly/WHOdoingwell**

## Recommendation 2: Support your patients

We know that people experiencing chronic illness, pain, loss of function or disability are more likely to have associated mental health problems (comorbidities). This can often have a negative effect on health behaviours and hinder recovery. Unless asked, people will not generally discuss mental health problems, but are more likely to do so if this forms a part of the consultation. Including mental health and wellbeing as part of routine practice can improve health outcomes and allow people to benefit from more comprehensive care that improves their overall wellbeing and quality of life. During emergencies, mental conditions become more common.^3^ Experience from Ebola Virus Disease outbreaks shows that epidemics have a particularly significant impact on individuals and communities,^4^ so this is even more important during the period of the COVID-19 pandemic.

### What you can do

- Ask about your patients how they are feeling. The World Health Organization (WHO) has developed the Psychological First Aid training package,^5^ which can be taught very quickly and help frontline staff to communicate empathically and respond in a considerate way if people are distressed or have signs of more significant mental illness. Visit **https://bit.ly/WHOfirstaid**
- In some services, for example when seeing people with more significant illness or disability, simple screening tools are available that have been widely validated and can help to identify people requiring further support.
- Put in place further support for people who need it. It is important that the small number of people who have more significant needs have access to a qualified person who can offer appropriate treatment, such as a psychiatric nurse, psychologist, psychiatrist or general doctor with mental health training. This may be via referral to a local mental health service or hospital. If this is not available, it is a good opportunity to advocate for such a service to be provided.

**“Health workers can be particularly vulnerable to anxiety and fear when providing treatment during an infectious disease epidemic. The social isolation imposed as a result of COVID-19 makes the situation even more challenging.”**

## Recommendation 3: Support other health care workers

Health care workers and other hospital staff members, including receptionists, cleaners and caterers, are particularly vulnerable to anxiety and fear when working during an infectious disease epidemic. It is important that managers recognise and address this so that staff members’ wellbeing can be protected.

### What you can do

- As a manager or employer, respond practically to people’s concerns, e.g., about adequate protection from infection during work, prevention of transmission to homes, economic stresses and the provision of adequate support to families and loved ones.
- All staff members should be able to have time off for breaks during periods of intense work (particularly if using personal protective equipment or if otherwise restricted in movement), and be given time and space to follow a healthy diet and take exercise.
- The workplace should be an environment where anxiety and stress can be discussed, and access to counselling support provided if possible.
